# Long-term, non-anthropogenic groundwater storage changes simulated by three global-scale hydrological models

**DOI:** 10.1038/s41598-019-47219-z

**Published:** 2019-07-24

**Authors:** Bailing Li, Matthew Rodell, Justin Sheffield, Eric Wood, Edwin Sutanudjaja

**Affiliations:** 1 ESSIC University of Maryland, Maryland, USA; 2 NASA Goddard Space Flight Center, Greenbelt, USA; 3 Princeton University, Princeton, USA; 4 University of Southampton, Southampton, England; 5 Utrecht University, Utrecht, Netherlands

**Keywords:** Climate and Earth system modelling, Hydrology

## Abstract

This study examined long-term, natural (i.e., excluding anthropogenic impacts) variability of groundwater storage worldwide. Groundwater storage changes were estimated by forcing three global-scale hydrological models with three 50+ year meteorological datasets. Evaluation using *in situ* groundwater observations from the U.S. and terrestrial water storage derived from the Gravity Recovery and Climate Experiment (GRACE) satellites showed that these models reasonably represented inter-annual variability of water storage, as indicated by correlations greater than 0.5 in most regions. Empirical orthogonal function analysis revealed influences of the El Niño Southern Oscillation (ENSO) on global groundwater storage. Simulated groundwater storage, including its global average, exhibited trends generally consistent with that of precipitation. Global total (natural) groundwater storage decreased over the past 5–7 decades with modeled rates ranging from 0.01 to 2.18 mm year^−1^. This large range can be attributed in part to groundwater’s low frequency (inter-decadal) variability, which complicates identification of real long-term trends even within a 50+ year time series. Results indicate that non-anthropogenic variability in groundwater storage is substantial, making knowledge of it fundamental to quantifying direct human impacts on groundwater storage.

## Introduction

Groundwater is crucial for meeting agricultural, industrial and municipal water needs, especially in arid, semi-arid and drought impacted regions where other types of fresh water are scarce^1^. Groundwater also sustains streams and rivers during dry periods through its contributions to baseflow^2^. Nevertheless, groundwater variations and their relationship to climate change are poorly understood at the global-scale due to the unavailability of observational data^3,4^. A few studies based on *in situ* observations have revealed the influence of large-scale climate signals such as the El Niño Southern Oscillation (ENSO) and Pacific Decadal Oscillation (PDO) on aquifers in the U.S.^5–8^ and Canadian Prairies^9^. Outside of North America similar studies are lacking because groundwater measurements typically are inaccessible because their distribution is restricted^10^. Further complicating matters, *in situ* observations are often unsuitable for climate studies because of short and discontinuous records, because they are made in confined aquifers where groundwater heads are controlled by factors other than climate or are affected by groundwater pumping^11^. The lack of knowledge about groundwater response to climate change has been noted in the 5^th^ assessment report (AR5) of Intergovermental Panel on Climate Change^12^ (IPCC).

It has been suggested that groundwater responses to future climate scenarios, at the global scale, will vary spatially, mainly depending on recharge rates, with arid and semi-arid areas more vulnerable than wetter areas such as the northern high latitudes, where recharge may increase^4^. Regional-scale studies support these predictions. A study found that projected changes in precipitation and temperature did not have significant impacts on groundwater recharge in the Grand Forks aquifer of British Columbia, Canada^13^. Another study showed that groundwater recharge in the drier and warmer part of the High Plains Aquifer exhibited higher sensitivity to rainfall changes than in the cool and wetter part of the aquifer and that the rate of changes in groundwater recharge amplified that of changes in precipitation^14^. Similarly, it was predicted that climate change would reduce recharge in the southern and mountainous parts of the western U.S., while recharge would be largely unchanged in the northwestern U.S^15^. These studies suggest that land evapotranspiration (ET), in addition to precipitation, may be an important factor controlling groundwater responses to climate change. Nevertheless, they are mainly focused on precipitation and temperature as the drivers of groundwater change, without considering other factors such as wind and air pressure for which changes are difficult to generalize due to internal variability and regional variation^16^. As a slower component of the hydrological cycle, groundwater may reflect small but persistent changes in the climate system^8^. To better predict the future, we need to improve our understanding on how groundwater has responded to changes that have occurred in the climate system over the past several decades. Such studies are noticeably lacking and limited to small spatial^17^ or short temporal scales^18,19^.

In this study, we investigate variations in global groundwater storage simulated by three global-scale hydrological models, the Catchment land surface model^20^ (CLSM), the WaterGAP^21^ and PCRaster Global Water Balance^22^ (PCR-GLOBWB) water resource models. CLSM is a physics based model that was developed for coupled land and atmospheric modeling including seasonal weather forecasting at NASA Goddard Space Flight Center with an emphasis on land-atmosphere fluxes, which are constrained by both energy and water balances. WaterGAP and PCR-GLOBWB are two leading models developed for assessing the spatial distribution and temporal variability of water resources around the world. They rely on empirical relations to compute all water budget components including surface water and human water use which are neglected by most land surface models including CLSM.

Forced with multiple decades of meteorological data that reflect changes in the climate system, these models are able to generate spatially and temporally continuous groundwater estimates suitable for studying climate change impacts on groundwater at regional to global scales^21,23–25^. Multi-model analysis also helps to reduce uncertainties associated with model physics and meteorological forcing fields, as trends in terrestrial water storage simulated by different models have been shown to vary considerably^26^. Modeled groundwater storage was evaluated using long-records of *in situ* data in the U.S. and modeled terrestrial water storage (TWS) was evaluated using TWS derived from Gravity Recovery and Climate Experiment (GRACE) satellites.

Because CLSM does not simulate human impacts such as groundwater abstraction, this study focuses on the temporal variability of natural (excluding anthropogenic impacts) groundwater storage changes associated with atmospheric effects (precipitation and ET). However, groundwater output from WaterGAP and PCR-GLOBWB with human water use is also analyzed in order to assess the uncertainty associated with evaluation using *in situ* and satellite data which may be affected by groundwater abstractions and to provide insight on the relative contributions of natural and anthropogenic groundwater variability to the total groundwater variability as groundwater depletion caused by abstraction has gained wide attention^1,21,27^.

## Data and Method

### Models

CLSM, which was developed at NASA Goddard Space Flight Center, simulates water storage changes in the surface layer (0 to 2 cm below the surface), the root zone (0 to 100 cm), and the full soil profile, and in three snow layers^20^. Water transfers among these stores are governed by empirical parameters derived based on topography, soil properties and average moisture transfer rates within different profiles. Profile depths are determined by CLSM parameter, bedrock depth, which varies spatially from 3 to 6 m and has direct impacts on the dynamics of simulated TWS and groundwater storage, with deeper bedrock generally producing slower groundwater responses to atmospheric drivers (precipitation and ET)^28^. The bedrock depth was increased worldwide by 2 m in this study to better simulate the full dynamic range of terrestrial water storage, particularly during severe drought, following previous studies^28–30^. No other parameters were adjusted or calibrated for this study. CLSM does not explicitly model water table variations but groundwater storage can be computed by subtracting root zone water storage from total soil profile storage.

CLSM accounts for sub-grid scale heterogeneity in soil wetness, including the fractions of the land surface where the soil is at or below the wilting point and transpiration has ceased, where the soil is unsaturated and above the wilting point and where it is saturated, to better simulate ET and runoff. Modeled state and flux updates occur at a 20 min time step to accommodate their highly non-linear relationships. CLSM simulated ET has been shown comparable with other land surface models in terms of capturing mean annual ET and its spatial patterns^31,32^. CLSM was run at 0.25° spatial resolution for this study.

The WaterGAP model (version 2), which was developed at the Centre for Environmental Systems Research of the University of Kassel, Germany, simulates water storage changes in the soil (with a single layer representing effective root depths depending on landcover type), groundwater, snow and surface water^21^. Water exchanges among these states and with the atmosphere are represented by simple empirical relations that facilitate model calibration and tuning. This version of WaterGAP was calibrated using mean annual streamflow data from more than 1,300 river basins covering more than 50% of the global land area. Outside of these basins, model parameters were estimated through regionalization. Benefiting from the simple (more linearized) model physics, the model was run on larger time steps (daily, compared to 20 min of CLSM) and coarser spatial resolution, 0.5°.

WaterGAP includes options to simulate anthropogenic effects such as reservoir operation and withdrawals from surface waters and groundwater. Water demands from all sectors (irrigation, livestock, industry and households) and net withdrawals are calculated based on the water use efficiency of each country or county, population development, and industrial and energy output. These are then input to the underlying hydrological model, along with atmospheric forcing data (precipitation, temperature and potential ET). Return flows are calculated as the difference between water inputs and net withdrawals. Anthropogenic groundwater storage estimates used in this study are based on an assumption of 70% irrigation efficiency, which is the ratio of consumptive water use to abstractions^21^.

PCR-GLOBWB has a model structure similar to that of WaterGAP. It also employs relatively simple empirical relationships to partition precipitation into surface runoff and infiltration and to simulate water transfers among different water stores, including surface water^22^. The model was not calibrated but its parameterizations were evaluated using GRDC runoff data. Sub-grid heterogeneity on land cover is also considered for calculating fluxes. PCR-GLOBWB can simulate anthropogenic effects but takes a different approach to estimating consumptive irrigation water use. Irrigation water demand is simulated by wetting soils to their field capacity in irrigated cells (or fractions of cells) during growing seasons. The estimated water demand is then subtracted from surface waters or groundwater. As a result, its simulated irrigation water demands depend heavily on model physics such as those for ET. Similar to WaterGAP, groundwater recharge in PCR-GLOBWB can come from the upper soil (either natural recharge or irrigation return flow), or from river-beds. Output used in this study is based on model simulation at 5 arc minute spatial resolution and a daily time step.

### Meteorological forcing data

The CLSM simulation was driven by the Princeton forcing dataset which comprises time series of precipitation, short and long wave radiation, air temperature, relative humidity, surface pressure and wind speed. The dataset, which spans 1948 to 2014, was constructed by bias correcting the precipitation, solar radiation, and air temperature fields from the National Center for Environmental Prediction (NCEP)/National Center for Atmospheric Research (NCAR) re-analysis using *in situ* and satellite observations^33^. The data are provided on a 1° global grid (excluding the Antarctica) with a 3-hourly timestep. The Princeton dataset has been used for a wide variety of applications including examining long-term trends of drought^34^, runoff^35^, and changes in the water cycle^36^. CLSM was first spun up from uniform initial conditions over the period, 1948 to 2014. The first 10 years of that simulation were discarded and the temporal (1959–2014) mean of each variable at each grid pixel on December 31 was used to initialize the experimental simulation on January 1, 1948. Use of multi-year average initial conditions is optimal for reducing the impacts of climatological anomalies on an experimental model simulation^37^.

WaterGAP simulations from 1960 to 2009 were driven by gridded time series of monthly precipitation and potential ET. Monthly precipitation obtained from Global Precipitation Climate Center^38^ (GPCC), Version 6, were downscaled by evenly distributing the monthly values to wet days of the month determined from Climate Research Unit (CRU) data^39^, version TS 3.10. CRU temperature and cloud cover data were used to derive monthly potential ET based on the Priestley and Taylor equation^40,41^ which requires fewer atmospheric variable inputs than the Penman-Monteith method used by most land surface models including CLSM. Monthly time series of potential ET were further downscaled to daily values prior to model simulation.

The forcing data used for PCR-GLOBWB simulations during 1958–2015 were based on monthly CRU (TS 3.21.2 for 1958–2010 and TS.3.24 for 2011–2015) precipitation, temperature and potential ET, which were spatially and temporally downscaled using ERA40 and ERA-Interim data^22^. CRU data are provided at 5 arc minute resolution but the temperature data were further downscaled for better simulation of snow dynamics. Monthly CRU precipitation was proportionally distributed to ERA rainy days for a given threshold (0.1 mm day^−1^) with daily values re-scaled to match monthly CRU precipitation. In areas with too few CRU stations, CRU precipitation was replaced by ERA precipitation, thus this precipitation dataset is referred to as CRU-ERA in this study. Monthly reference ET was estimated outside of the model simulation using CRU meteorological data and the Penman-Monteith method and was downscaled to daily values using a daily temperature-based ET product. PCR-GLOBWB was first repeatedly executed for 150 years using the mean atmospheric forcing conditions under the non-anthropogenic scenario from 1968–2000 to allow simulated groundwater to reach a steady state. The resulting conditions were then used to initialize the model under non-anthropogenic and anthropogenic simulation scenarios.

### *In situ* and GRACE satellite data

*In situ* groundwater observations were obtained for the four Mississippi sub-basins and four northeastern U.S. regions (see Supplementary Fig. S1) from the USGS web site. These 181 wells were selected because they are located in unconfined or semi-confined aquifers, are not directly affected by groundwater withdrawals or injections and have been continuously monitored for 10–30 years. These data have been used in previous studies for validating GRACE TWS^11,42–44^ and investigating scale-dependency of groundwater storage^45^. Depth-to-water measurements were converted to groundwater storage anomalies using individual estimates of specific yield for each well following published procedures^11,28^.

The GRACE satellites mapped Earth’s gravity field on a monthly basis from 2002 to 2017. The resulting time variable gravity observations were used to derive TWS anomalies which reflect changes in soil moisture, groundwater, snowpack, and surface water storage (lakes and rivers). GRACE also detected water storage changes associated with anthropogenic effects such as groundwater abstractions which, along with surface water, are not simulated by CLSM. The GRACE data used in this study were provided approximately monthly during the mission period on a 0.5° global grid. They were developed using a regularization method that eliminates the need for post-processing and thus better preserves TWS amplitudes and trends^46^. Despite the grid resolution, the effective resolution of this GRACE product is still around 150,000 km^2^ due to the limitations of the method used^47,48^.

For comparison with *in situ* data, modeled groundwater storage at the grid cell nearest each well was selected. Because this study focuses on large-scale interannual variability, monthly precipitation, ET, groundwater storage and TWS were averaged to 1° spatial resolution and monthly anomalies were calculated by removing the long-term mean of each calendar month. Anomalies were further standardized (thus unitless) so that they had zero mean and standard deviation of one. Annual anomalies are averages of the standardized monthly anomalies in each year. Unless otherwise noted, anomalies discussed in following sections refer to standardized anomalies.

### Methods

Regular Empirical Orthogonal Function (EOF) analysis^49^ was applied to annual anomalies (weighted by area) of Princeton forcing data, CLSM ET estimates, GPCC and CRU-ERA precipitation, and simulated groundwater storage to identify patterns of variability and their relationship with large-scale climate signals. The non-parametric Mann-Kendall method^50^ was used to calculate linear trend and quantify changes over the simulation periods.

## Results

### Evaluation of model estimates

**Figure 1:**
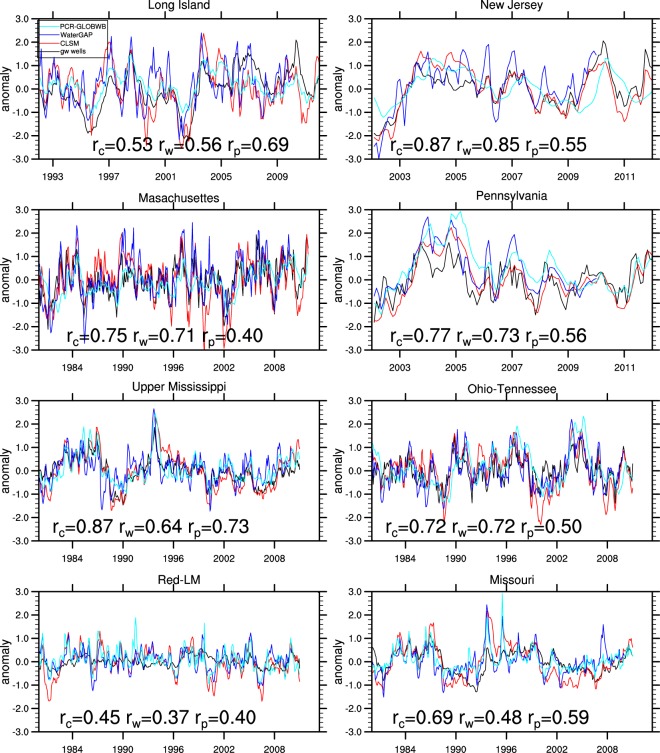
Standardized monthly groundwater storage anomalies from *in situ* observations and CLSM, WaterGAP and PCR-GLOBWB under the non-anthropogenic scenario for the four northeastern U.S. regions and the four sub-basins of the Mississippi river. “r” represents correlation between *in situ* observations and modeled groundwater with subscribes c, w and p representing CLSM, WaterGAP and PCR-GLOBWB, respectively.

Figure 1 shows that the correlation coefficient between groundwater storage anomalies simulated by the three models and observations generally exceeds 0.5. The exception is the combined Red River and Lower Mississippi (Red-LM) region, where the mean water table depth (around 17 m) is much deeper than other regions^28^; as a result, groundwater response to atmospheric effects is considerably lagged and may not be well represented by the simplified groundwater schemes in these models. PCR-GLOBWB groundwater lags and shows considerably lower correlation with *in situ* data than the others in New Jersey, Massachusetts, Pennsylvania and Ohio-Tennessee.

Groundwater is a significant source of fresh water in some of these regions and thus it is possible that certain wells were directly affected by groundwater withdrawals, despite our effort to exclude such wells. Supplementary Figs S2 and S3 show that simulating withdrawals had more persistent impacts on groundwater in Long Island where groundwater is heavily abstracted for municipal water supplies^51^ and Red-LM where groundwater is abstracted to support irrigated agriculture^21,22^, but it did not always improve the correlation with *in situ* observations in these two and other regions. These results suggest that the chosen observation wells outside of Long Island and Red-LM are generally unaffected by pumping and that simulation of withdrawals does not guarantee improved correlation even where withdrawals are significant. Simulating groundwater abstractions are challenged by lack of information on irrigation water demand, efficiency, timing and duration of irrigation^21^.

Across the models, the correlation between WaterGAP and CLMS natural groundwater output exceeds 0.5 in most land areas except the Arabian Peninsula, the Amazon, high-mountain Asia and high latitudes of North America (see Supplementary Fig. S4). In drier climates where the latency between atmospheric phenomena and groundwater responses is large, discrepancies in model physics and precipitation data may enhance the differences in simulated groundwater. In regions where snowmelt is a major source of groundwater recharge, differences in simulated snow pack among models^25^ translate to lower correlation among simulated groundwater time series. CLSM does not represent surface water and its associated recharge, resulting in lower correlation with models that do (WaterGAP and PCR-GLOBWB) in humid tropical and boreal river basins in which surface water is a significant part of TWS^52^. CLSM groundwater correlation with PCR-GLOBWB resemble that with WaterGAP (see Supplementary Fig. S5) including those areas of low correlations. WaterGAP and PCR-GLOBWB do not simulate natural groundwater storage changes in the Sahara Desert, hence the zero correlation between their groundwater and that of CLSM in that area.

**Figure 2:**
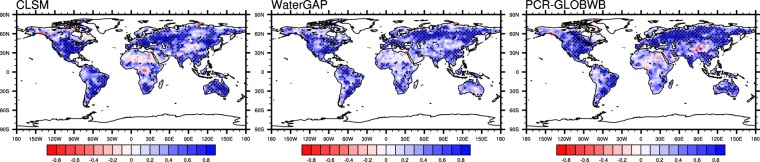
Correlation between monthly TWS anomalies from GRACE and those from CLMS, WaterGAP and PCR-GLOBWB under the non-anthropogenic scenario. Staples indicate correlation greater than 0.5.

To help overcome the lack of spatial coverage of well data, modeled TWS was compared with GRACE TWS. Figure 2 shows that modeled monthly non-anthropogenic TWS anomalies generally correlate well with the observations, with correlation coefficients exceeding 0.5 in large areas of North America, South America, Northern Eurasia, Southern Asia and Australia (for CLSM and PCR-GLOBWB only). In northern Africa and northwestern China, the correlations are low, possibly due to the scarcity of *in situ* observations for bias correcting and calibrating forcing data. Correlation is also reduced in regions with significant groundwater depletion such as Northern India^10^, which was detected by GRACE but not represented in these simulated non-anthropogenic TWS. Similarly, rapid glacier and ice cap melt in the Gulf of Alaska^53^ and the Canadian archipelago^54^ are also not simulated, causing poor correlations in those areas. Negative correlation in the Congo basin may be attributed to lack of surface water in CLSM. Despite their representation of surface water, WaterGAP and PCR-GLOBWB do not always show better correlation with GRACE TWS than CLSM in large river basins such as the Amazon and the Nile (Fig. 2), underscoring the challenge of simulating TWS and its components. As noted previously, despite the 0.5° resolution of the GRACE TWS product we used, the effective resolution of GRACE is still around 150,000 km^2^ ^47,48^ and thus small scale TWS variability not resolved by GRACE may contribute to diminished correlation with the models. Indeed, CLSM TWS exhibits much higher correlation (>0.6) with GRACE TWS when averaged over large basins^25^.

Simulating human water use in WaterGAP and PCR-GLOBWB led to improved correlation with GRACE TWS (see Supplementary Figs S6 and S7) in most regions where excessive groundwater withdrawals have led to groundwater depletion such as Northern India, the Middle East and the North China Plain. In the Great Plains, recharge from ephemeral playas may lead to overestimation of irrigation water requirement in these models^21^ and hence degraded correlation with GRACE TWS. Overall, simulating withdrawals and water management changed the correlation of simulated TWS and with GRACE TWS by more than 0.02 in about 10% of the 1° grid cells over the global land area (excluding Antarctic and the Greenland,). This highlights the importance of evaluating non-anthropogenic TWS using GRACE data as in Fig. 2.

### Modes of variability

**Figure 3:**
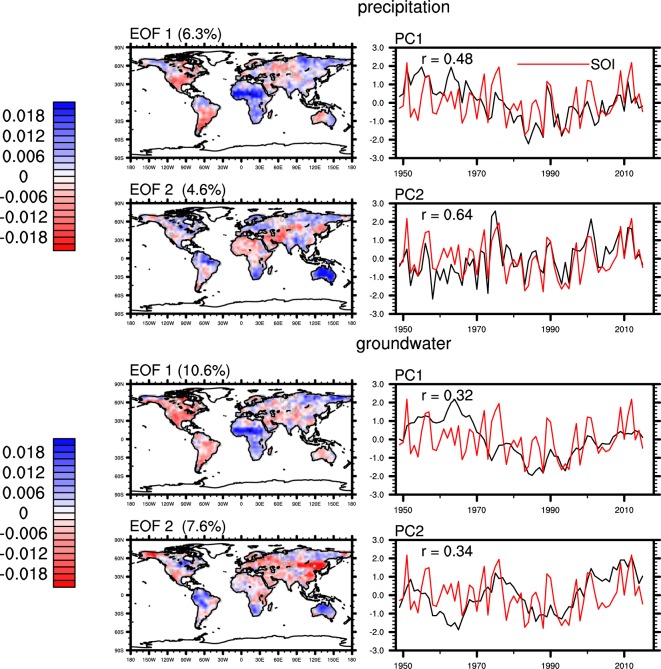
Spatial and temporal patterns of the first two EOFs of annual standardized Princeton precipitation and CLSM groundwater storage anomalies. Numbers in parentheses represent percentages of variance explained over total variance. Red lines represent annual SOI. “r” represents the correlations between normalized PCs and SOI which are significant at the 0.01 level.

Empirical Orthogonal Function (EOF) analysis has been widely used to find important patterns of variability in climate datasets^55,56^. By construction, EOF analysis yields modes of variability, consisting of a spatial pattern and a temporal component (or principal component), with their explained variance over the total variance in decreasing order^57^. Natural variability of groundwater is mainly governed by precipitation and ET^45,58^. Thus, we first examine modes of variability of global precipitation and ET. Figure 3 shows that the principal component (PC) of the leading mode of variability in Princeton precipitation, PC1, is significantly (significance levels are provided at figure captions) correlated with the Southern Oscillation Index (SOI). PC1 contains a ‘trough’-shaped low-frequency variability, i.e., decreasing from 1948 to mid-1980 and increasing afterwards which is also observed in SOI. The spatial pattern of EOF 1 shows the low-frequency variability is mainly associated with changes in the Sahel, which experienced a multi-decadal drought starting from 1960^59^. EOF 2 identified the low latitude regions where ENSO is known to have stronger impacts on precipitation, namely, Australia, Southern Africa and Northern South America^56^. Indeed, PC2 of Princeton precipitation exhibits higher correlation (0.64) with SOI. These areas, including the Sahel, were also identified in a previous study^60^ which showed stronger correlation (0.82) between PC1 of global precipitation and ENSO, likely due to the longer period (1900–1982) of that study.

**Figure 4:**
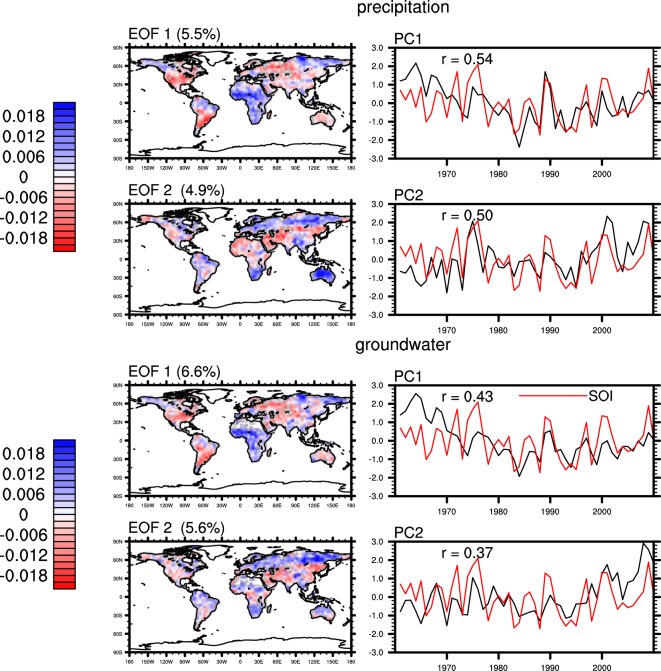
same as Fig. 3 but for annual standardized GPCC precipitation and WaterGAP groundwater storage anomalies.

**Figure 5:**
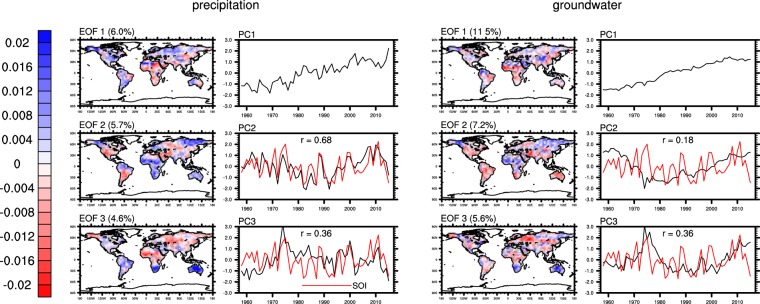
Same as Fig. 3 but for the first three EOFs of annual standardized CRU-ERA precipitation and PCR-GLOBWB groundwater storage anomalies. Correlations are significant at the 0.01 level except that between PC2 of groundwater and SOI.

The leading modes of variability in global GPCC precipitation (used as input to WaterGAP) exhibit spatial and temporal patterns (Fig. 4) similar to those of Princeton precipitation, including the low frequency variability in PC1. In contrast, EOF 1 of CRU-ERA precipitation (used as input to PCR-GLOBWB) is an apparent trend (Fig. 5), mainly associated with changes in the Sahara Desert, Alaska, the high-mountains of Asia and the southern Arabian Peninsula. Its EOF 2 and 3 exhibit spatial patterns similar to EOF 1 and EOF 2 of Princeton and GPCC precipitation. EOF analysis of CRU precipitation produced similar results (not shown) as those of Princeton and GPCC precipitation, suggesting that the trend in EOF1 of CRU-ERA precipitation was caused by replacing CRU precipitation with ERA estimates at locations with few CRU gauges.

ET can have a significant impact on shallow groundwater and a warm climate would tend to favor increased ET. Supplementary Figs S8 and S9 show that the leading modes of variability in Princeton longwave radiation, air temperature, relative humidity, air pressure and wind are apparent trends, presumably reflecting climate change^61,62^. In contrast, EOF 1 of global CLSM ET is similar to that of Princeton precipitation (see Supplementary Fig. S10). Both display the trough-shaped variability in PC1, suggesting strong control of precipitation on ET. EOF 2 of global CLSM ET is an apparent trend associated with increasing ET in northern Eurasia, the northeastern U.S. and Central Australia, and decreasing ET in northern South America and the Sahel. This trend only accounts for 7.3% of the total variance while most of the leading modes in the atmospheric forcing fields account for more than 20% of the total variance (see Supplementary Figs S8 and S9). PCR-GLOBWB and WaterGAP ET output was not analyzed because the methods and high-resolution data used by those models to downscale potential and reference ET may also impact modes of variability in actual ET estimates, making it difficult to interpret the results.

The leading modes of variability in CLSM and WaterGAP groundwater show spatial and temporal patterns similar to those of their corresponding precipitation inputs (Figs 3 and 4). The PCs of CLSM groundwater contain less high-frequency variability than those of Princeton precipitation, reflecting the low-pass filtering effect of groundwater recharge^63^. PC1 of CLSM groundwater exhibits the largest change in 1960s and 1970s, when PC2 of ET and PC1 of precipitation also exhibit their largest changes. These are mainly attributed to significant decrease in precipitation in the Sahel and increases in ET in northern Europe, the northeastern US and central Australia during that period. PCs of groundwater from both CLSM and WaterGAP show lower correlation with SOI than that between precipitation and SOI, reflecting the influence of other processes and fluxes on groundwater such as ET.

On the other hand, the leading modes of variability in PCR-GLOBWB groundwater bear less resemblance to those of CRU-ERA precipitation. Its EOF 1 is still a trend, but its spatial pattern differs from that of EOF 1 of CRU-ERA precipitation, suggesting other factors may have had significant impacts on simulated groundwater. Similarly, its EOF 2 does not resemble that of CRU-ERA precipitation while its EOF 3 shows spatial and temporal patterns similar to EOF 3 of CRU-ERA precipitation.

### Long-term trends

**Figure 6:**
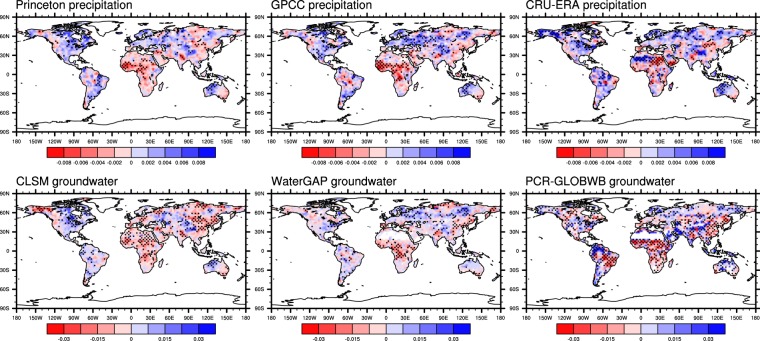
Mann-Kendal trend of annual standardized Princeton, GPCC, CRU-ERA precipitation (top row), and CLSM, WaterGAP and PCR-GLOBWB non-anthropogenic groundwater storage anomalies (bottom row). Stipples represent significant trend at the 0.05 level.

Figure 6 shows the Mann-Kendal trend of annual standardized precipitation and non-anthropogenic groundwater storage anomalies from the three models. As in the EOF analysis, Princeton and GPCC precipitation datasets exhibit similar trends including strong decreases in the Sahel and sub-tropical Africa and increases in the northeastern US and Western Europe, changes also reported in IPCC/AR5^16^. In contrast, CRU-ERA precipitation exhibits different trends, especially in the Sahara Desert, Alaska and the Amazon, most of which were identified in its EOF 1 (Fig. 5).

The CLSM and WaterGAP groundwater trends resemble their corresponding precipitation trends in most land areas, particularly in much of Africa, eastern Asia and Northern Russia, where groundwater decreased significantly due to below normal precipitation. In the Hudson Bay and the Great Lake regions, the two models produced different trends in groundwater. This may be attributed to the fact that CLSM does not simulate surface water and its related recharge. Decreasing CLSM groundwater in much of northern Europe appears to result from increasing ET (see Supplementary Fig. S11), and from a combination of ET increases and precipitation decreases in east-central Asia. Northern Eurasia experienced decreased surface air pressure and, in some areas, increased wind speed (see Supplementary Fig. S12), both of which favor increased ET and thus decreased groundwater storage. Changes in surface air pressure in the high latitudes were noted in IPCC AR5^16^. Several re-analysis products also show increased storminess in northern, western, and central Europe towards the end of the 20^th^ century, more so in the North Sea and Baltic Sea regions^64^. More frequent cyclones (associated with a low pressure center) in the lower arctic Canada in 1953–2002 were also reported^65^. Since WaterGAP estimates potential ET using the Priestly-Taylor equation, which does not require wind and air pressure data, its estimated ET likely would not reflect these changes.

As in other analyses of this study, PCR-GLOBWB groundwater trends exhibit spatial patterns different from those of CLSM and WaterGAP (Fig. 6). PCR-GLOBWB groundwater increased significantly in high altitude regions of northern South America and Iran, despite no associated increases in CRU-ERA precipitation. The model uses lapse rates derived from a high-resolution temperature climatology data to downscale CRU temperature data in order to better simulate snow dynamics. It is possible this downscaling scheme led to earlier and increased snowmelt, and hence increased groundwater recharge in those areas.

**Figure 7:**
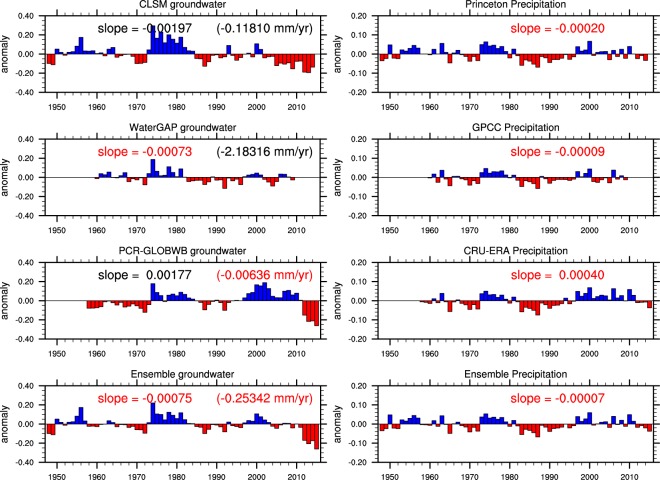
Global-averaged non-anthropogenic groundwater storage anomalies from the three models and their ensemble average (left column) and their corresponding global precipitation (right column). Slopes are Mann-Kendal trend (numbers in black indicate significant trend at the 0.05 level). Numbers in parentheses are trend of non-standardized global total groundwater storage anomalies.

Figure 7 shows that globally, CLSM and WaterGAP groundwater storage (ignoring anthropogenic effects) decreased during their respective simulation periods, consistent with the trends in their corresponding global precipitation datasets. Both time series also exhibit low frequency variability, namely a change in the mid-1980s from generally positive to generally negative anomalies, resulting in negative trends. Apparently, ET did not contribute to the negative trend in CLSM groundwater because global averaged ET was relatively stable over the simulation period with an insignificant decreasing trend (see Supplementary Fig. S13). Changes in other atmospheric forcing fields such as increased humidity and air pressure and decreased wind speed (see Supplementary Fig. S14) may suppress the effect of rising temperature on ET. The global standardized CLSM groundwater time series has larger amplitude variation than that of WaterGAP (Fig. 7), owing to larger amplitude variations in global Princeton precipitation than in global GPCC precipitation, and hence a larger (and significant) decreasing trend. In contrast, Global PCR-GLOBWB groundwater exhibits an increasing trend, consistent with the trend in global CRU-ERA precipitation (Fig. 7).

Groundwater responds to surface wet and dry conditions asymmetrically^58^, e.g., an intense rainfall event in which the water runs off rapidly may do less to replenish an aquifer than a slow, soaking rain. Moreover, groundwater levels reflect the cumulative effects of atmospheric conditions, so that one rainy month or year may not be enough to return groundwater levels to normal after a drought, for example. Differences in model physics such as those controlling partitioning of precipitation into surface runoff and infiltration may further amplify these asymmetrical responses and discrepancies among the models. This is why the three global groundwater storage time series exhibit weaker cross-correlations than the global precipitation time series (Table 1). In the case of this study, global PCR-GLOBWB groundwater was consistently the least well correlated among groundwater output of the three models (Table 1).

**Table 1:** Cross-correlation between global total groundwater storage of the three models (Fig. 7) and between global total precipitation of the three datasets.

	CLSM & WaterGAP	CLSM & PCR-GLOBWB	WaterGAP & PCR-GLOBWB
global groundwater	0.67	0.48	0.47
	Princeton & GPCC	Princeton and CRU-ERA	GPCC and CRU-ERA
global precipitation	0.91	0.90	0.86

The standardized anomalies discussed above enable examination of modes of variability and comparison of trends across different regions. Figure 7 also shows the trends of global, non-standardized annual groundwater storage anomalies (in mm year^−1^) from three models which range from −0.01 to −2.18 mm year^−1^. Note that the trend in global non-standardized PCR-GLOBWB groundwater is negative, in contrast to the positive trend in the standardized time series. This is because groundwater storage anomalies in dry climate may exhibit significant trend but their contributions to the global total non-standardized groundwater storage anomalies (mm) are diminished due to their small amplitude variations (mm).

For comparison, global total groundwater by WaterGAP and PCR-GLOBWB, including anthropogenic processes, decreased significantly (Figs S15 and S16). Global standardized PCR-GLOBWB groundwater exhibits much smaller decreasing (0.001 vs 0.002 from WaterGAP) trend, owing to the increasing trend in global PCR_GLOBWB non-anthropogenic groundwater. EOF 1 in the global anthropogenic groundwater is a trend (see Supplementary Figs S17 and S18) mainly associated with decreases in known regions of intensive groundwater abstractions such as the Arabian Peninsula, Northern India, the North China Plain and the Great Plains^21^. Its remaining EOFs reflect the influence of climate variability, also identified in the corresponding non-anthropogenic groundwater output (Figs 4 and 5). Once again, WaterGAP and PCR-GLOBWB produced drastically different rates of decline in global non-standardized anthropogenic groundwater, 46.15 and 0.35 mm year^−1^, respectively (see Supplementary Figs S15 and S16).

## Discussions and Conclusions

This study investigates the long-term variability of global non-anthropogenic groundwater storage and its environmental controls. Due to the scarcity of *in situ* groundwater observations, we analyzed simulated groundwater storage from the Catchment land surface model (CLSM), WaterGAP and PCR-GLOBWB water resource models. The outputs from these three models may be considered independent given that the model parameterizations are quite distinct and each model was driven by a different atmospheric forcing dataset. Modeled groundwater storage and terrestrial water storage (TWS) were evaluated using *in situ* data in the eight U.S. regions and GRACE derived TWS at the globe scale. The three output datasets compared reasonably well with the observations based on correlations greater than 0.5 in most regions, demonstrating skill in reproducing interannual variability of water storage. Output from the models was somewhat consistent, especially between CLSM and WaterGAP, owing to the more consistent precipitation data used by those two models.

Neglecting anthropogenic impacts, global total groundwater storage decreased during the past 50–70 years, at a rate of 0.25 mm year^−1^ based on the ensemble average. It is possible that the long-term decline is a symptom of climate change, but considering that low-frequency variability in the global groundwater storage time series and in regional groundwater of deeper aquifers^45^, the 50 to 67 years of simulation periods may not be long enough to identify a secular trend with confidence. This low frequency variability together with the sensitivity of trends to anomaly amplitudes that vary among models may have also contributed to the discrepancies among trends estimated by the three models. Therefore, better characterizing that low frequency variability would be valuable for improving the models and interpreting the apparent groundwater storage trends.

The EOF analysis revealed that ENSO influenced the variability of global groundwater through its control of precipitation patterns. Because ENSO also exhibits low frequency variability, it is plausible that multidecadal scale variability in global total groundwater can be partly attributed to ENSO, though more research would be needed to describe those relationships.

Our analysis shows that simulated regional and global groundwater anomalies are sensitive to trends in precipitation. Therefore, the linear trend in global groundwater reported here must be interpreted in the context of the reliability and temporal consistency of the forcing datasets, which currently remain incompletely established. For the Princeton dataset, the effectiveness of bias-correction depends on regional availability of *in situ* data, and may rely more on the fidelity of the NCEP/NCAR re-analysis (used as a baseline) when and where *in situ* data are scarce. Meteorological data tend to be scarce in the deep tropics, at high latitudes and during earlier years of the 1948–2014 period, especially for fields such as pressure and wind speed^66^. The GPCC and CRU precipitation data are based on gauge observations and their spatial and temporal variability and consistency may be adversely affected by changes in the numbers and locations of gauges. On the other hand, simulated ET did not exhibit a significant trend and thus was not a factor in the simulated global groundwater depletion trend.

Our analysis shows that representing groundwater abstraction for supporting irrigated agriculture increased the magnitude of the decreasing trends in global groundwater storage simulated by WaterGAP and PCR-GLOBWB. In addition, groundwater depletions became the leading mode of variability in global groundwater, surpassing ENSO. However, the resulting trends in global groundwater storage, 46.15 to 0.35 mm year^−1^ for WaterGAP and PCR-GLOBWB, respectively, are directly impacted by non-anthropogenic trends and the low frequency variability in global WaterGAP and PCR-GLOBWB non-anthropogenic groundwater (Fig. 7) are embedded in their corresponding global anthropogenic groundwater time series (Figs S15 and S16). This emphasizes the importance of evaluating natural groundwater variability when assessing human impacts on groundwater resources. This is particularly true for models like PCR-GLOBWB which rely on the underlying hydrological model to estimate consumptive irrigation water use.

Finally, we caution that our evaluation using *in situ* groundwater data was limited to regions of the U.S. that do not represent the climates of Africa, east-central Asia, and Northern Eurasia, where some of the significant groundwater storage changes were simulated. Uncertainty in simulated groundwater time series may be higher in drier climates where aquifers tend to be deeper and therefore have longer-scale variability and disconnection from the atmosphere which are difficult to model. Evaluation using GRACE data is limited to less than 15 years when GRACE was active and provides no insight on a model’s ability to separate groundwater from other TWS changes.

## Supplementary information

Supplementary Information for the article
