# Supplementary material for: Long-term, non-anthropogenic groundwater storage changes simulated by three global-scale hydrological models
Source: Sci Rep. 2019 Jul 24;9:10746. doi: 10.1038/s41598-019-47219-z (PMC6656779; doi:10.1038/s41598-019-47219-z)
Supplement: Supplementary file 1 — Supplementary Information for the article [file 41598_2019_47219_MOESM1_ESM.pdf]

Supplementary Information

Long-term, non-anthropogenic groundwater storage changes simulated by three global-scale hydrological models

*Bailing Li [1,2], Matthew Rodell[2], Justin Sheffield[3,4], Eric Wood[3] and Edwin Sutanudjaja[5]

[1] ESSIC University of Maryland

[2] NASA Goddard Space Flight Center

[3] Princeton University

[4]University of Southampton

[5]Utrecht University

This file contains 18 figures, Figs.S1 to S18.

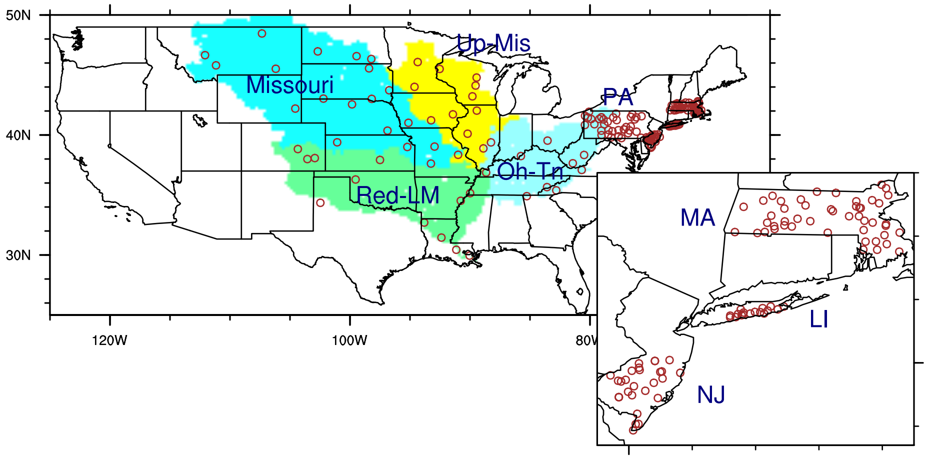

Fig. S1. Locations of groundwater observation wells in Pennsylvania ("PA"), Massachusetts ("MA"), New Jersey ("NJ") and Long Island ("LI") of New York and the four sub-basins of the Mississippi river, upper Mississippi ("Up-Mis"), Ohio-Tennessee ("Oh-Tn"), Missouri and the combined Red River and lower Mississippi basin ("Red-LM").

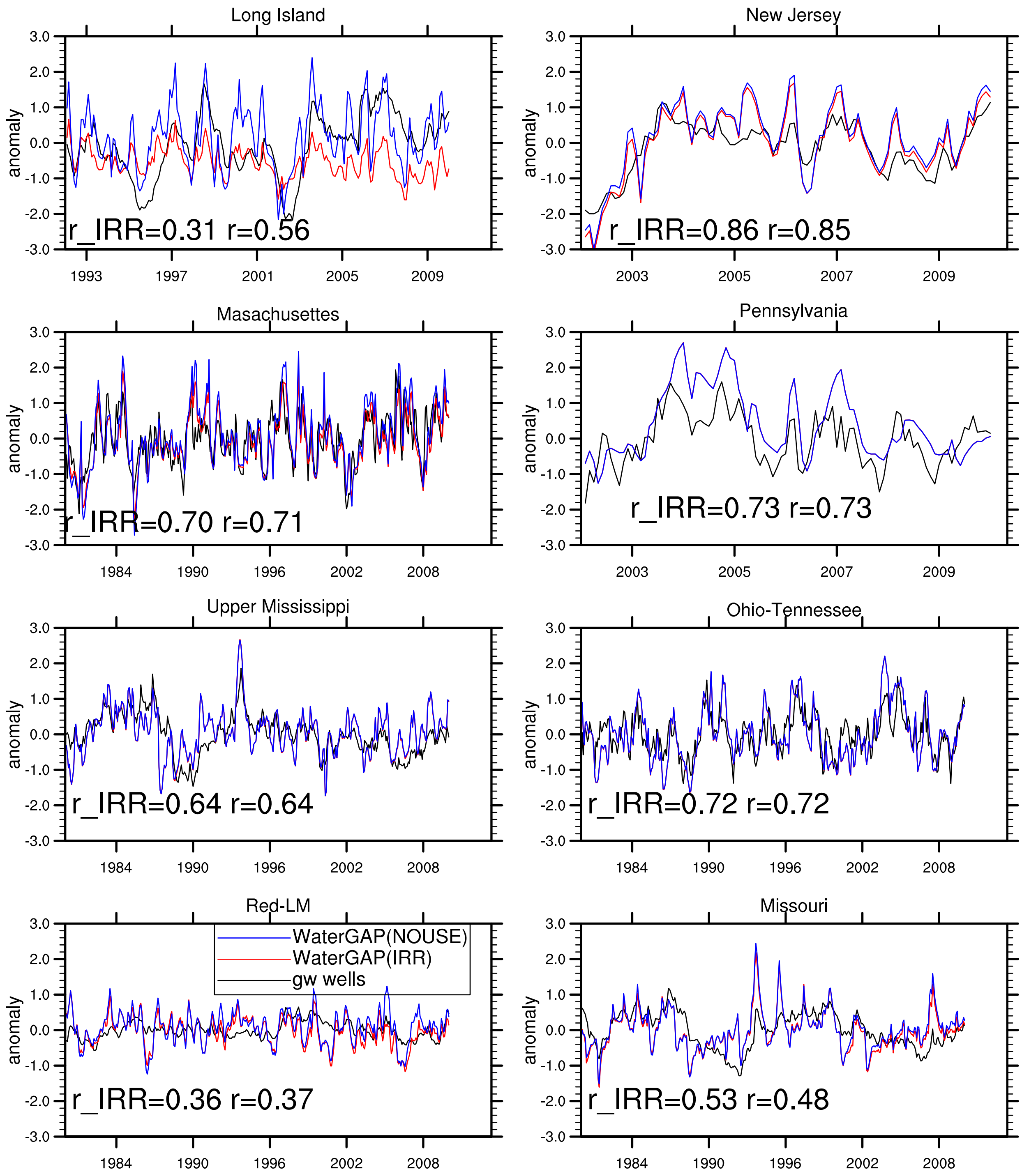

Fig. S2 Monthly standardized anomalies of in situ observations and WaterGAP groundwater storage under non-anthropogenic (NOUSE) and anthropogenic (IRR) scenarios. r and r_IRR represent correlations between in situ groundwater and modeled groundwater under the aforementioned two scenarios, respectively.

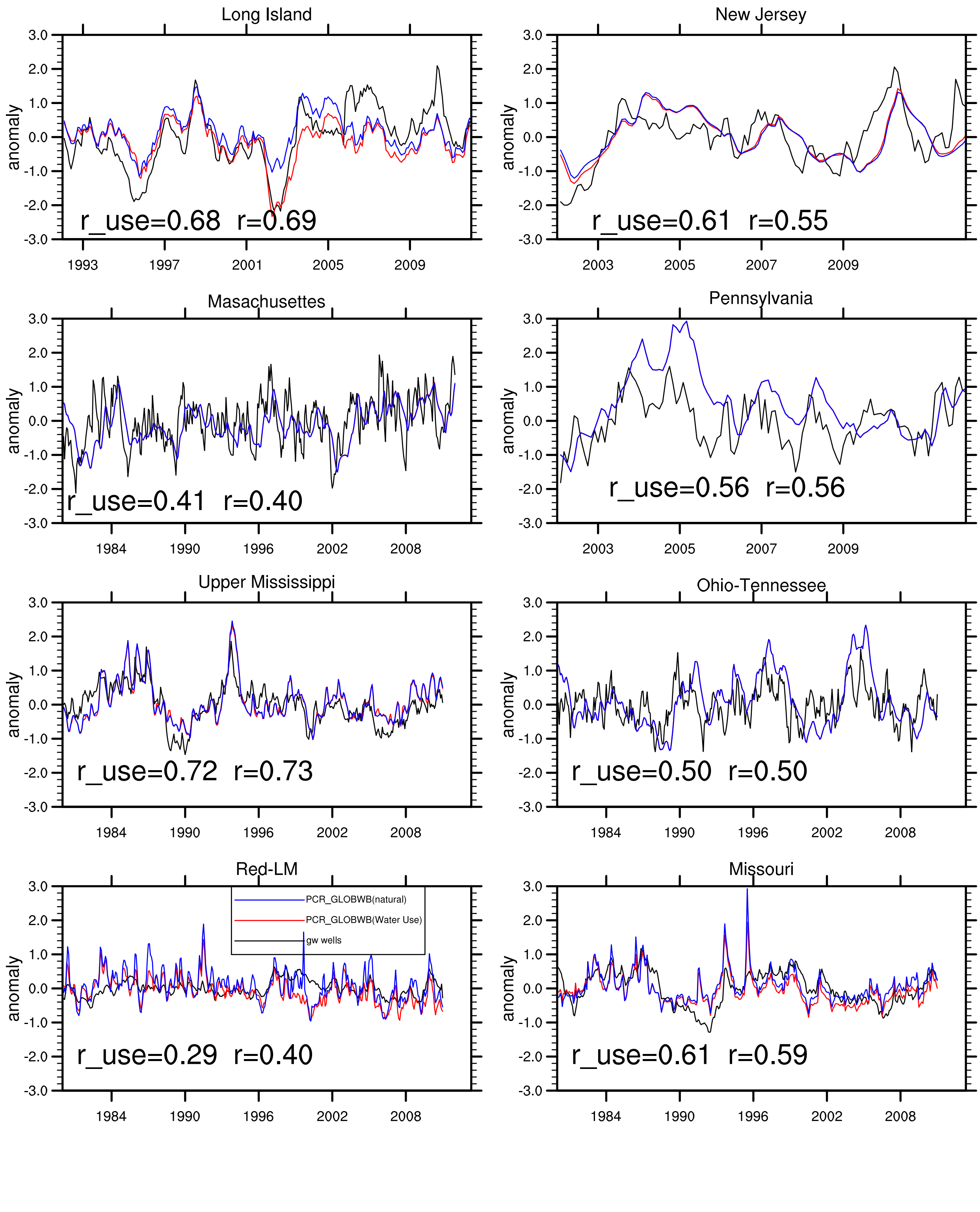

Fig. S3 Monthly standardized groundwater storage anomalies of in situ observations and those from PCR-GLOBWB under the natural and human water use conditions. r and r_use represent correlations between in situ observations and the two model output, respectively.

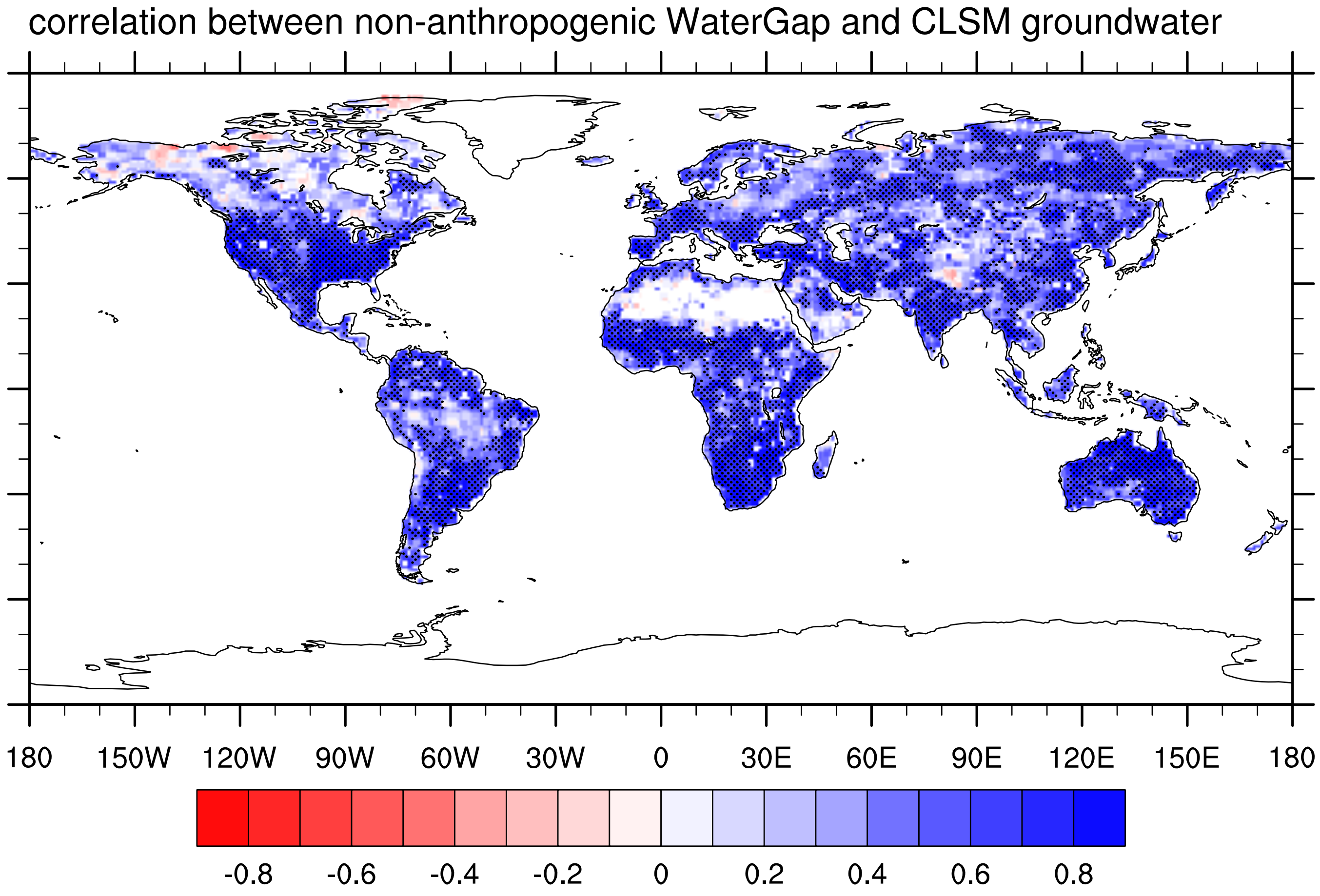

Fig. S4. Correlation between annual non-anthropogenic CLSM and WaterGAP groundwater storage anomalies.

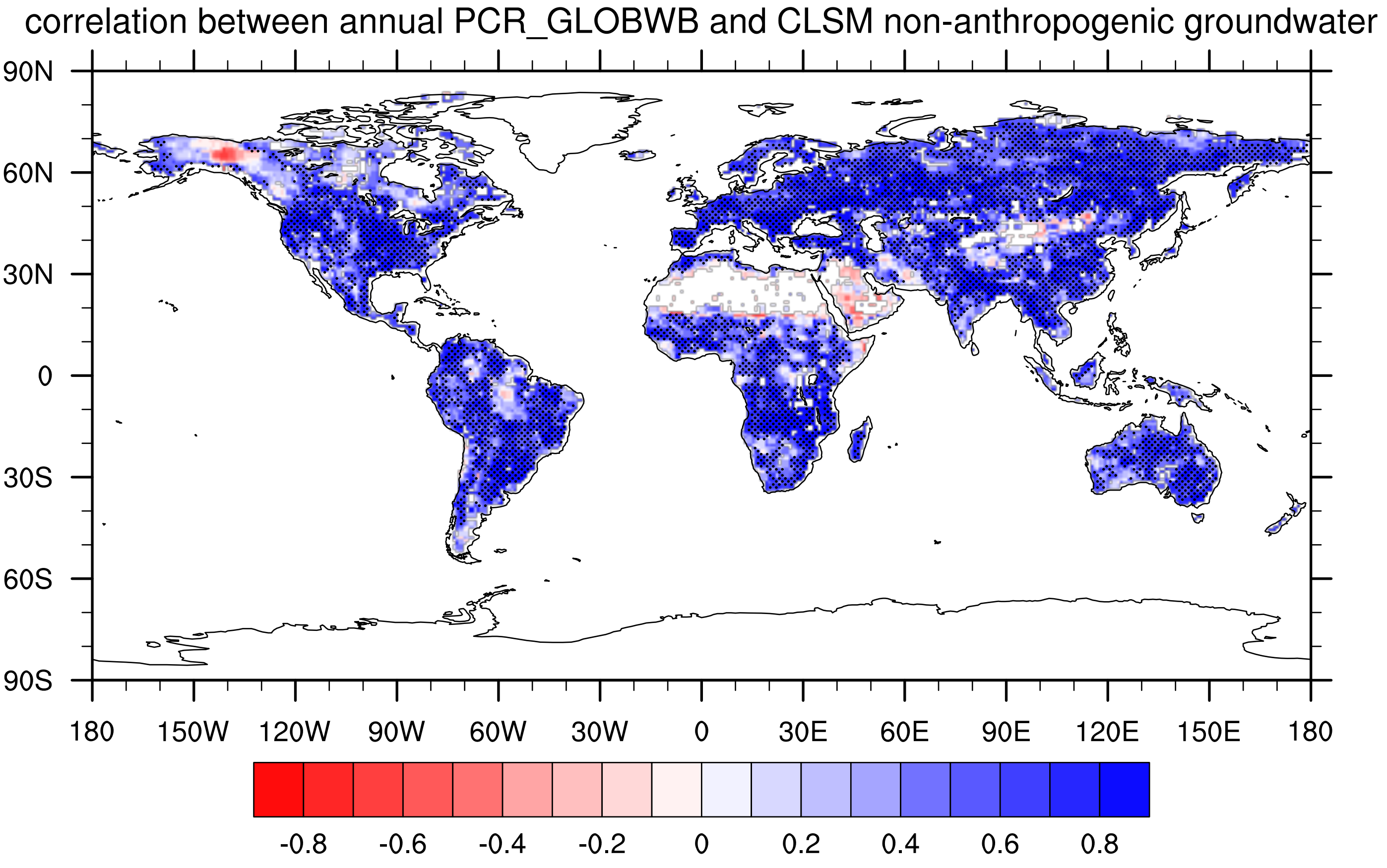

Fig. S5. Correlation between annual CLSM and PCR-GLOBWB non-anthropogenic groundwater storage anomalies.

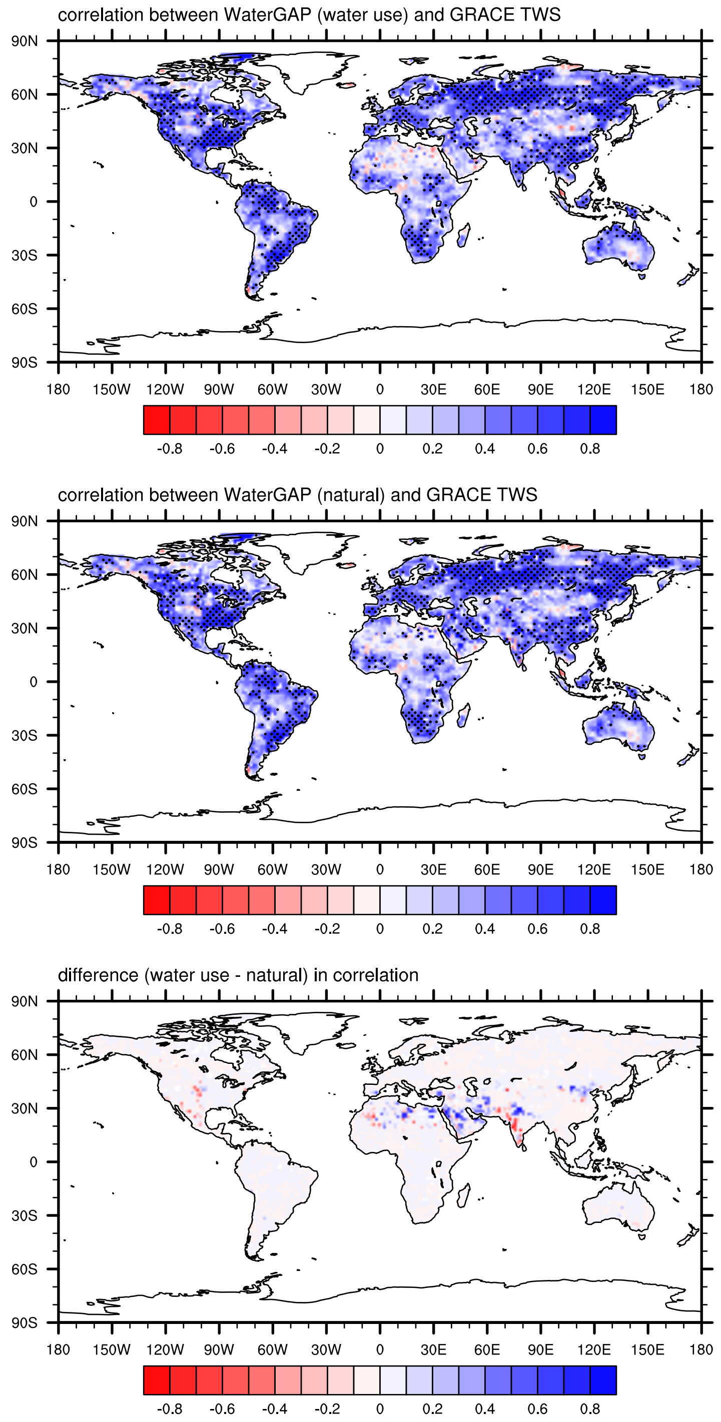

Fig.S6. Correlation between monthly GRACE and WaterGAP TWS under the anthropogenic (water use) and non-anthropogenic (natural) scenarios, respectively (upper two panels). The lower panel shows the difference between the upper two panels.

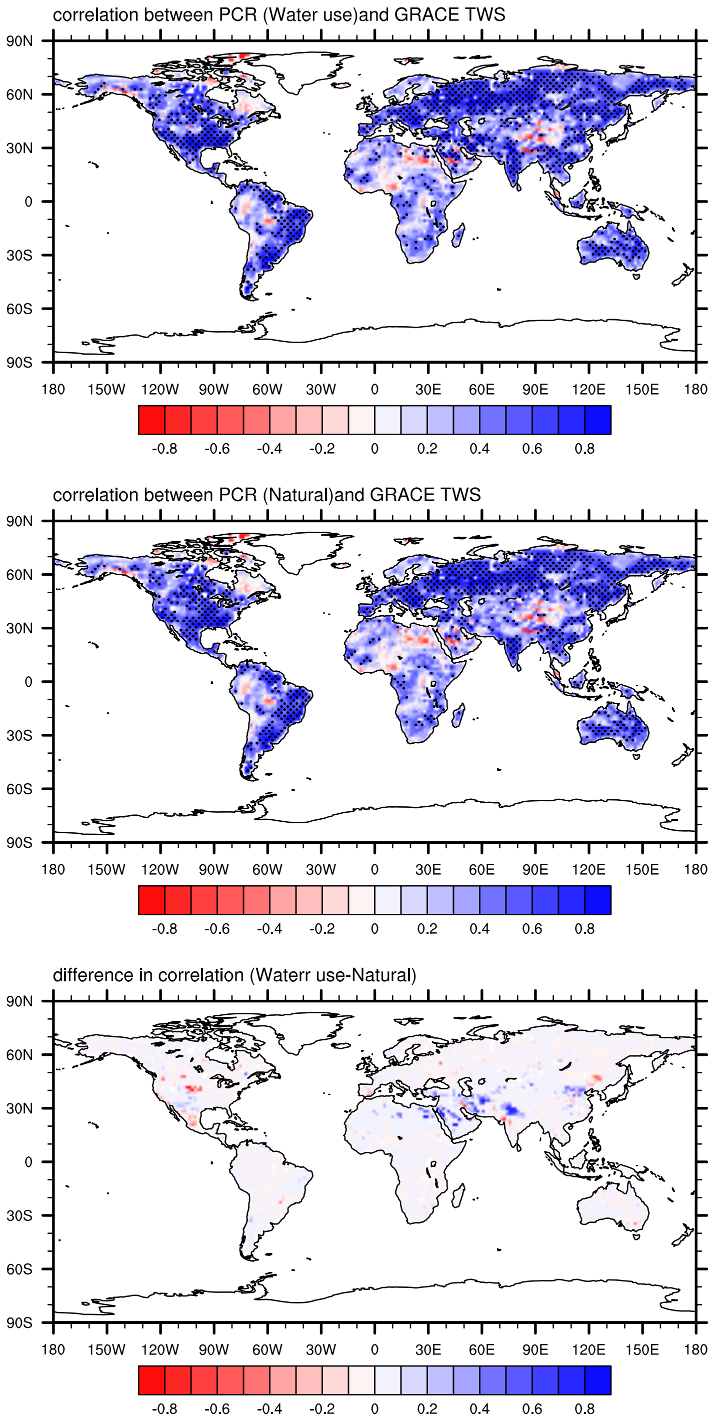

Fig.S7. Correlation between monthly GRACE and PCR-GLOBWB TWS anomalies under the human water use and natural conditions (upper two panels). The lower panel represents the difference between the upper two panels.

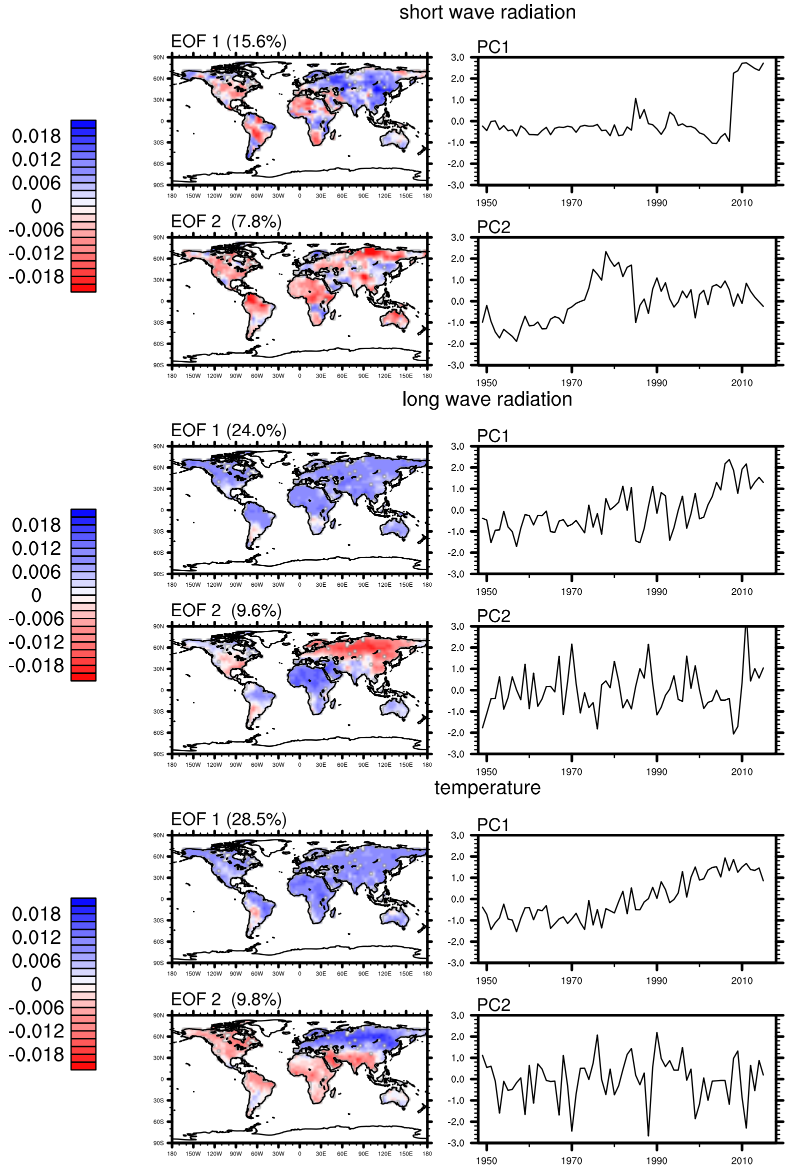

Fig. S8. Spatial and temporal patterns of the first two leading modes of variability for the annual anomalies of Princeton short and long wave radiation, air temperature.

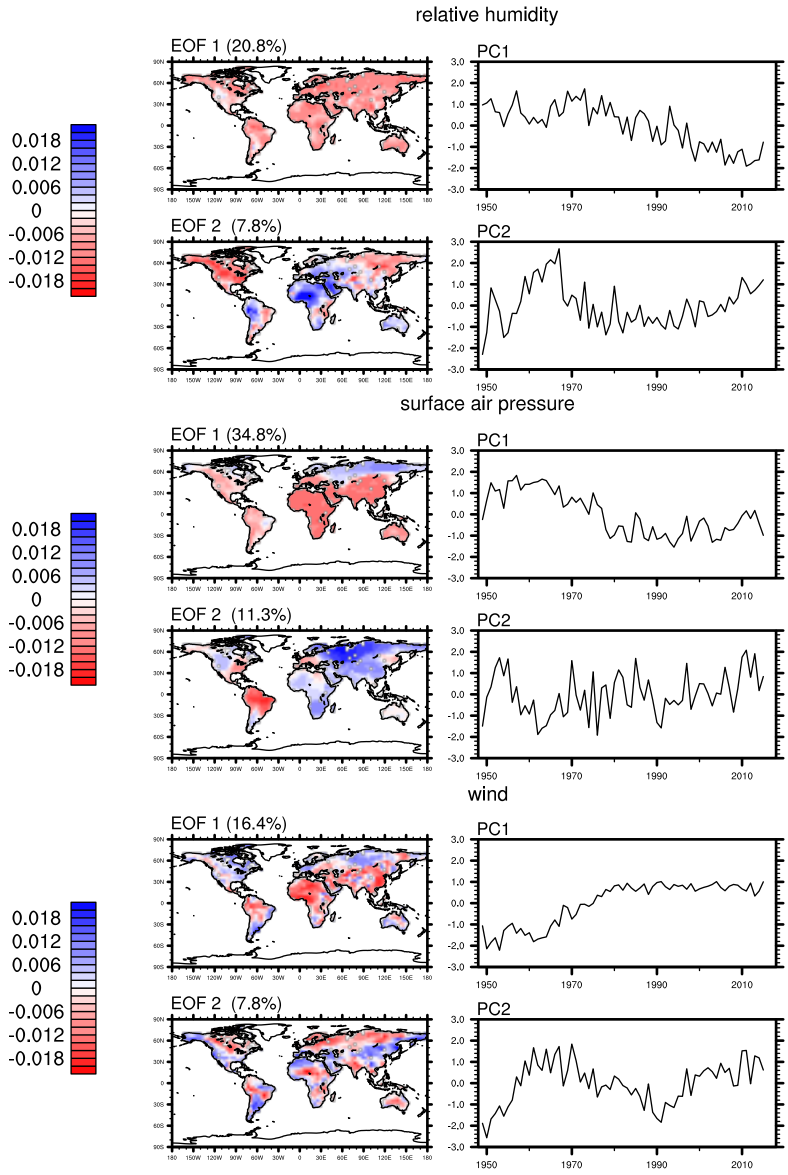

Fig. S9. Same as Fig. S8 but for relative humidity, surface air pressure and wind.

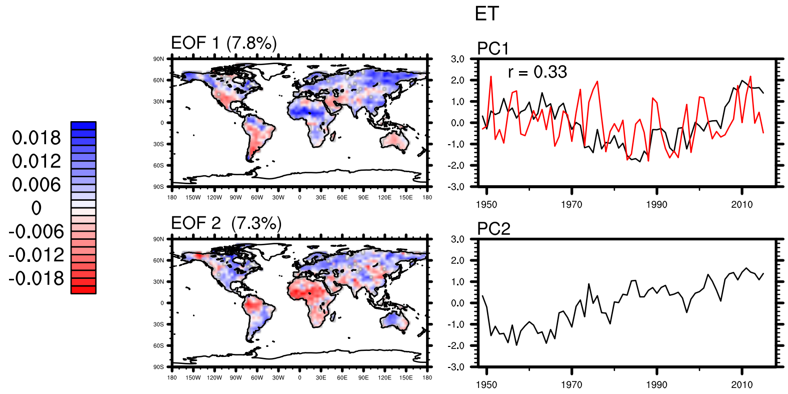

Fig. S10. Spatial and temporal patterns of the first two leading modes of variability of annual standardized CLSM ET anomalies. The red line represents SOI and r represents the correlation between SOI and PC1.

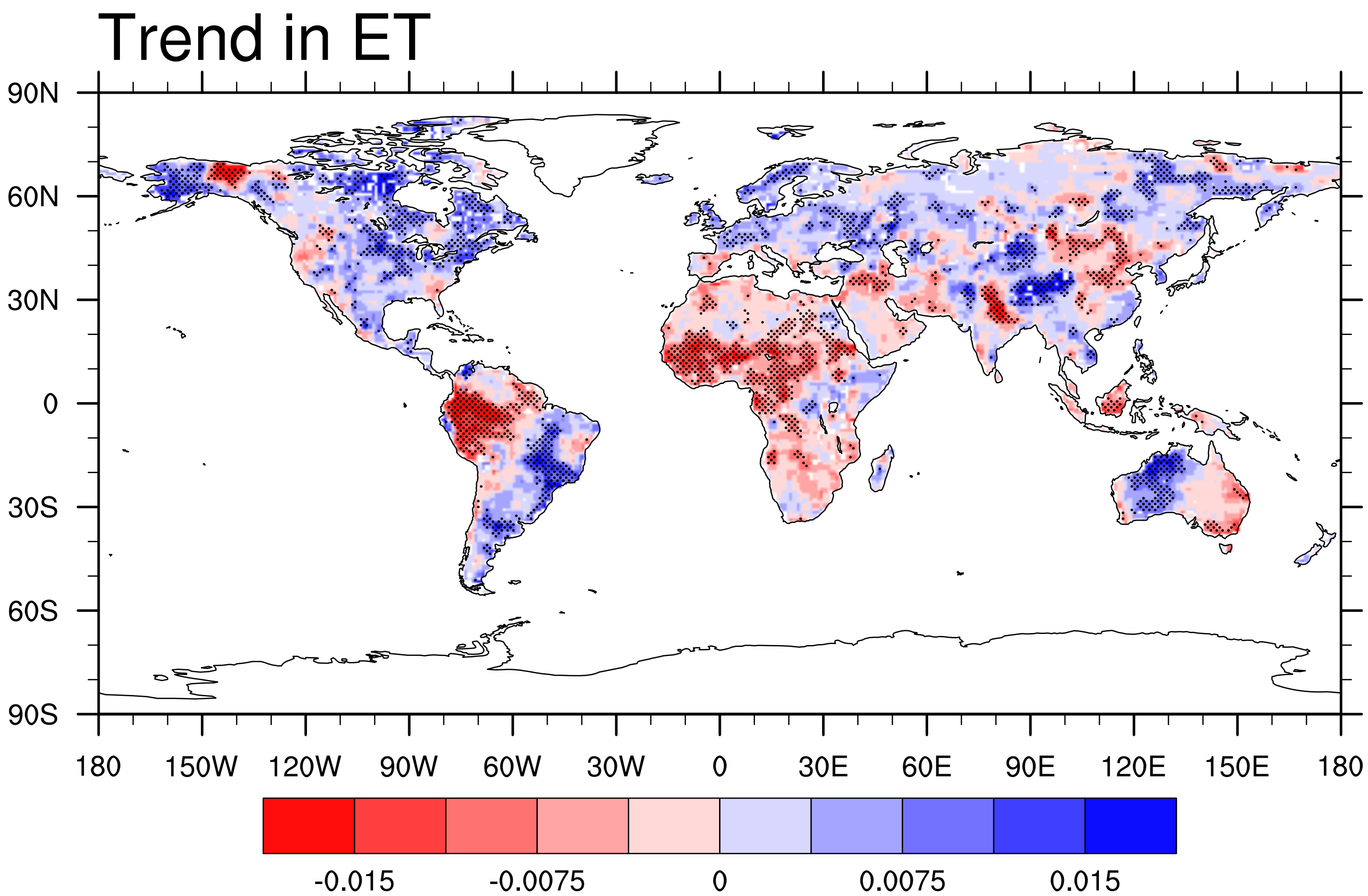

Fig. S11. Mann-Kendal trend of annual standardized CLSM ET anomalies for 1948-2014. Stipples represent significant trends at the 0.05 significant level.

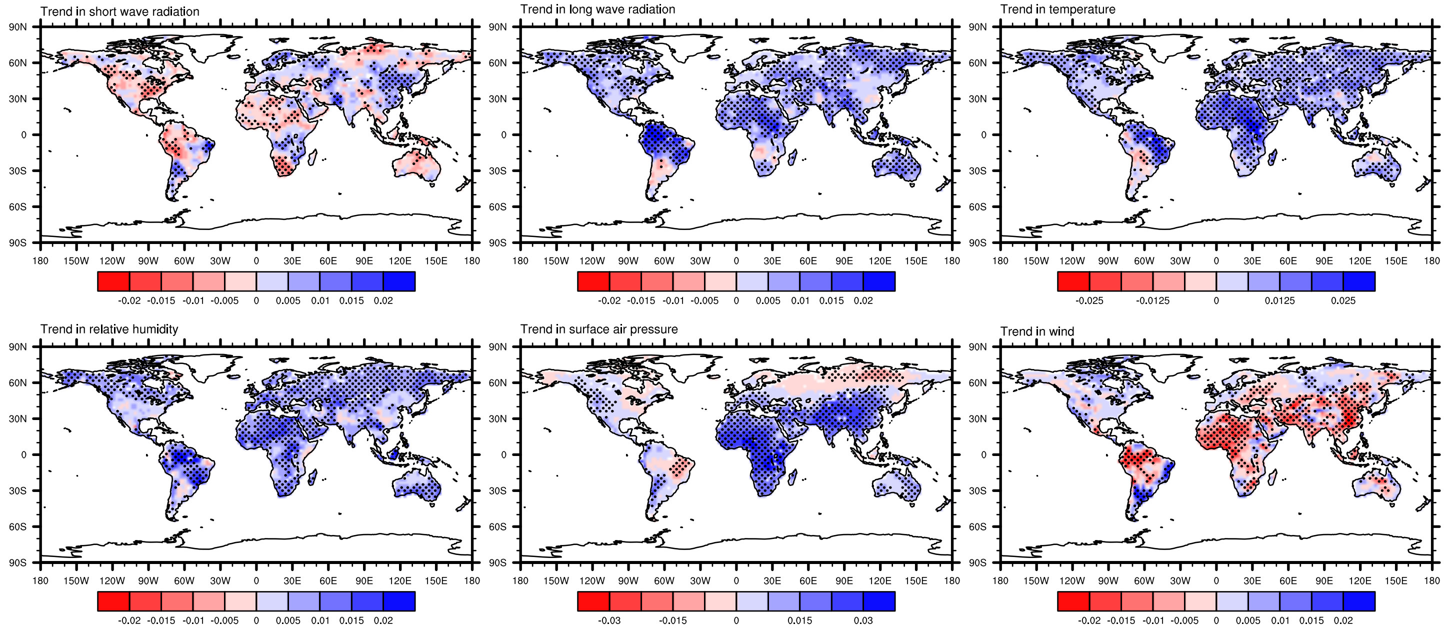

Fig. S12. Mann-Kendal trend of annual standardized anomalies of short and long wave radiation, temperature, relative humidity, surface air pressure and wind for 1948-2014. Stipples represent significant trends at the 0.05 significant level.

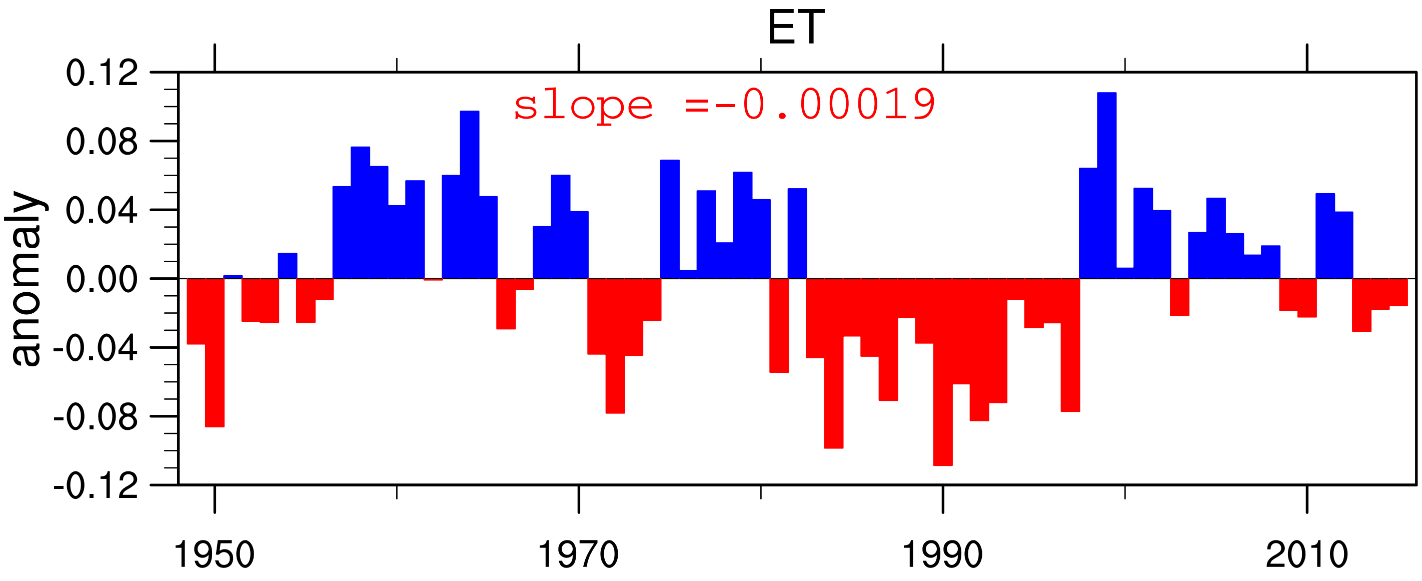

Fig. S13. Global averaged annual standardized CLSM ET anomalies. Slope is the Mann-Kendal trend (numbers in red represent statistically insignificant trend at the 0.05 level).

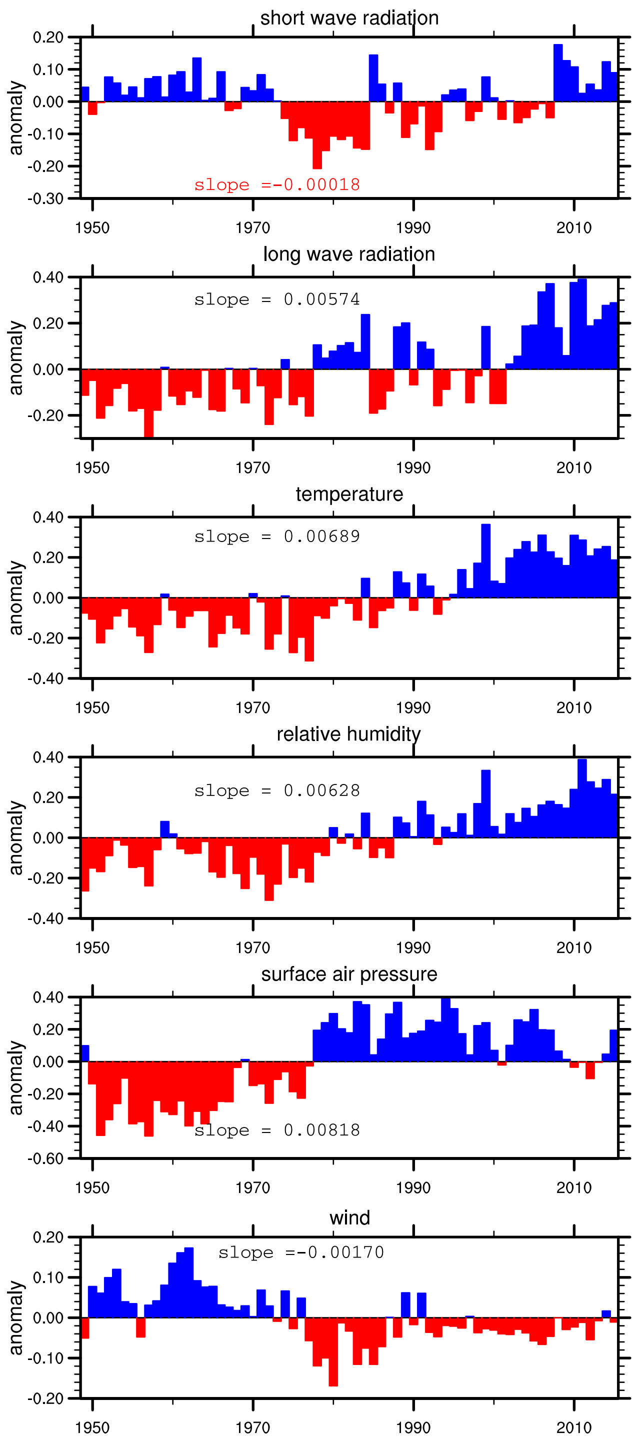

Fig. S14. Global averaged annual standardized anomalies of long and short wave radiation, temperature, relative humidity, surface air pressure and wind. Slopes are Mann-Kendal trend for each time series (numbers in red represent statistically insignificant trend at the 0.05 level).

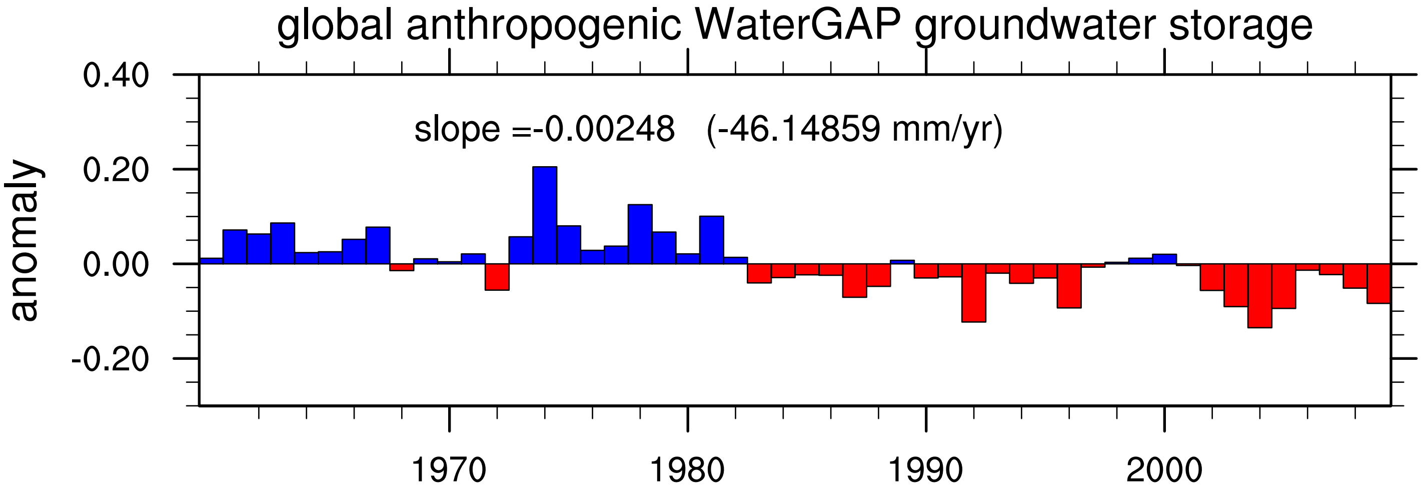

Fig.S15 Global total groundwater storage by WaterGAP under the anthropogenic scenario during 1960-2009. The number in parentheses represents trend in the non-standardized global anthropogenic groundwater anomalies.

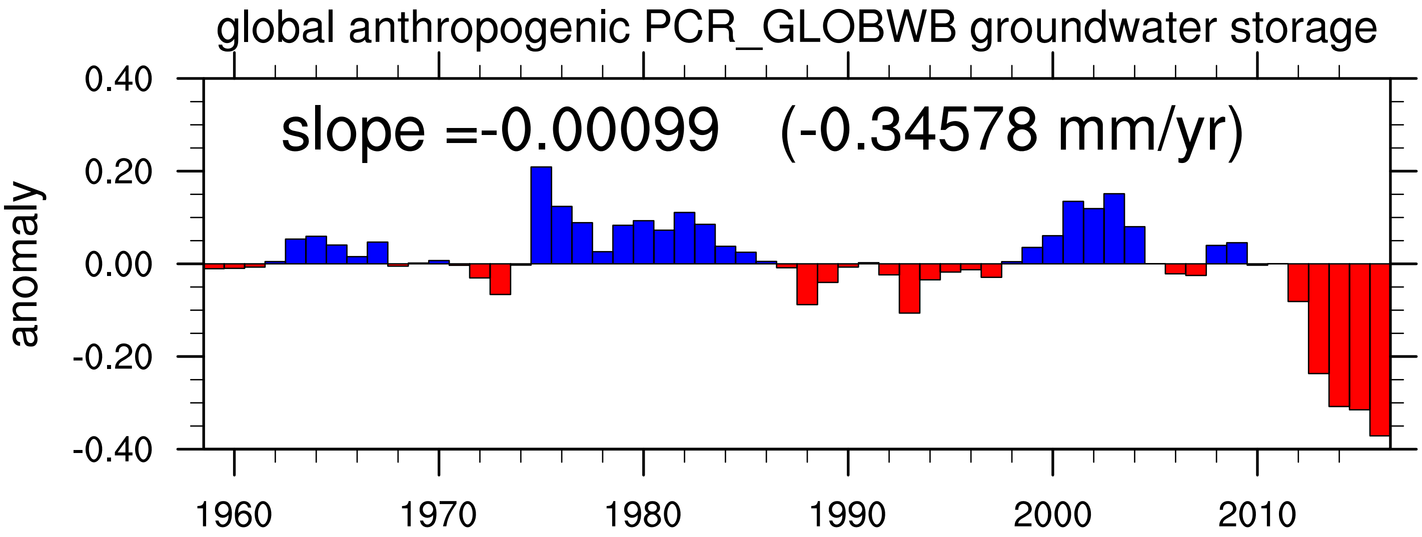

Fig.S16 Global total anthropogenic groundwater storage by PCR-GLOBWB during 1958-2015. The number in parentheses represents trend in the non-standardized global anthropogenic groundwater anomalies.

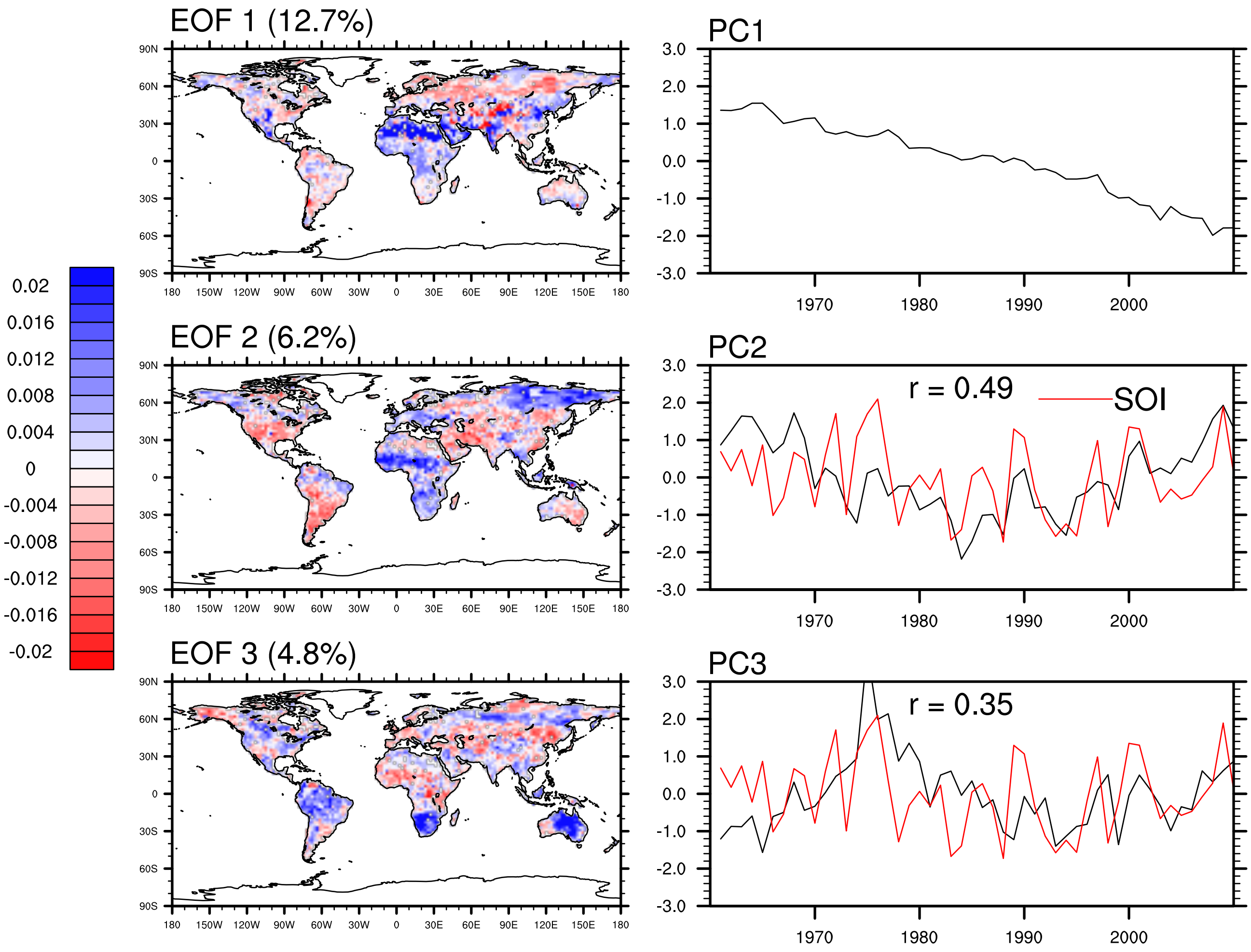

Fig. S17 Spatial and temporal patterns of the first three EOFs of annual standardized anthropogenic groundwater storage anomalies by WaterGAP.

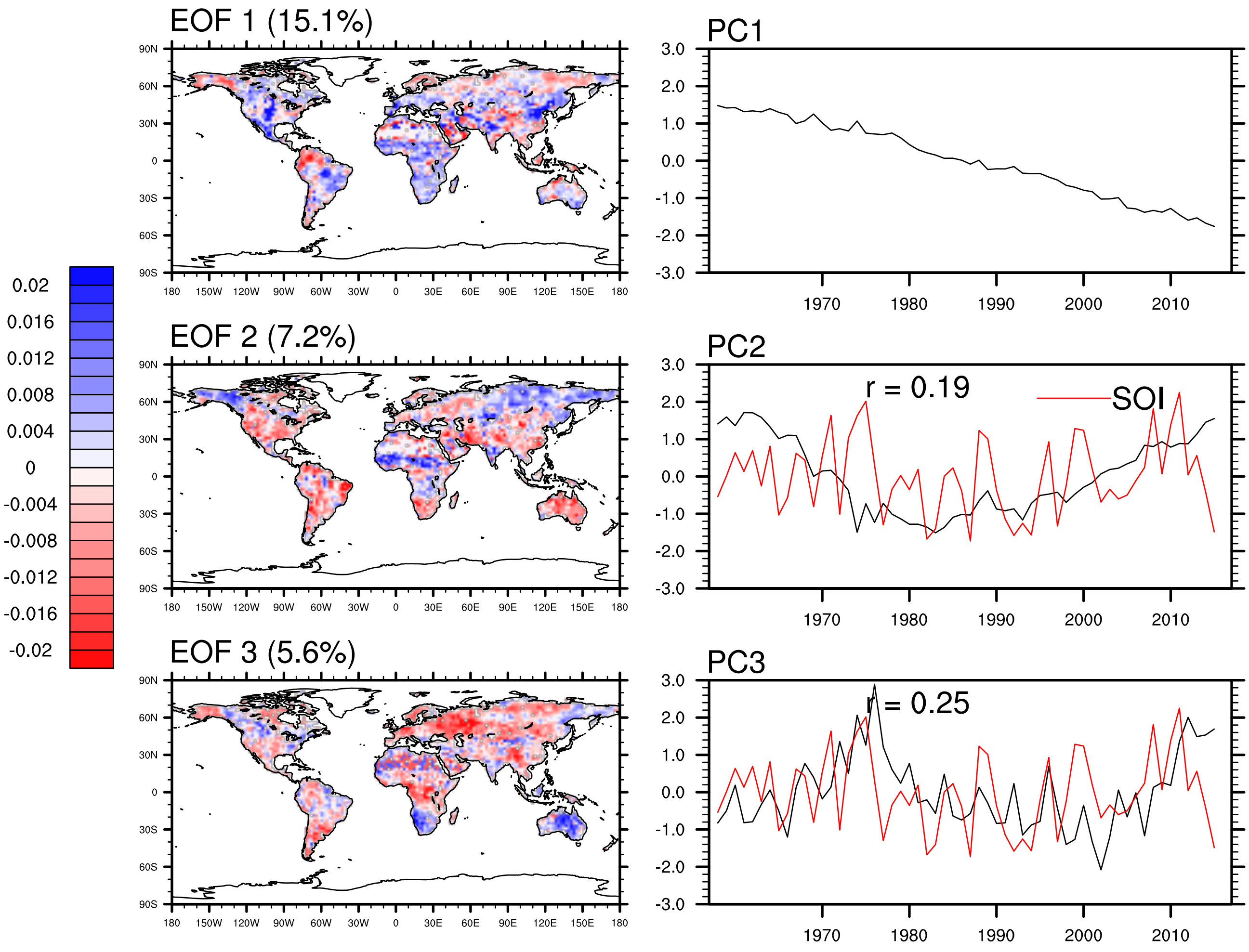

Fig. S18 Spatial and temporal patterns of the first three EOFs of annual standardized anthropogenic groundwater storage anomalies by PCR-GLOBWB.
